# Electro-assisted integration of nanodiamonds into conducting polypyrrole for functional coatings

**DOI:** 10.1038/s41598-025-31402-6

**Published:** 2025-12-13

**Authors:** Karolina Cysewska, Anita Stoppel, Muhammad Saqib, Birgit Paul, Julia Kristin Hufenbach, Joerg Opitz, Natalia Beshchasna

**Affiliations:** 1 Faculty of Electronics, Telecommunications, and Informatics, and Advanced Materials Centre, Gdansk University of Technology, ul. Narutowicza 11/12, 80-233 Gdańsk, Poland; 2 Fraunhofer Institute for Ceramic Technologies and Systems IKTS, Maria-Reiche Strasse 2, 01109 Dresden, Germany; 3 Leibniz Institute for Solid State and Materials Research (IFW) Dresden, Helmholtzstrasse 20, 01069 Dresden, Germany

**Keywords:** Conducting polymer, Degradable metal, Electrodeposition, Nanodiamond, Smart coating, Conjugated polymers, Biomedical engineering

## Abstract

This work presents a novel method for electrically integrating functionalized nanodiamonds (NDs) into smart conducting polymer polypyrrole (PPy) coatings on highly active FeMnC alloy surfaces for stent applications. The optimized electrodeposition process produces stable PPy-ND coatings with tunable compositions using passivating sodium salicylate and chemically functionalized NDs. Attenuated total reflectance Fourier transform infrared (ATR-FTIR) spectroscopy and energy dispersive X-ray (EDX) analyses confirm the successful integration of NDs into the polymer matrix. Concurrently, scanning electron microscopy (SEM) reveals distinct morphological features influenced by deposition parameters. The coatings exhibit tunable corrosion properties in preliminary studies, highlighting their relevance for degradable metallic implant applications. This innovative method facilitates the development of multifunctional coatings, providing a versatile platform for advanced bio-interfaces in medical implants. The study shows the feasibility of combining conducting polymers with nanomaterials to enhance surface functionality and tackle critical challenges in implantable device technologies.

**Supplementary Information:**

The online version contains supplementary material available at 10.1038/s41598-025-31402-6.

## Introduction

Cardiovascular diseases, particularly those caused by atherosclerotic lesions in cardiac or peripheral arteries, remain one of the leading causes of death worldwide. Endovascular stents are widely used to restore blood flow in obstructed vessels. However, despite the success of bare-metal stents, complications such as in-stent restenosis (ISR) and in-stent thrombosis remain significant clinical challenges, with recurrence rates of 15–30% within the first year post-implantation^1^. To mitigate these issues, polymer-coated drug-eluting stents (DES), releasing antimitotic agents like paclitaxel or rapamycin, were developed to inhibit smooth muscle cell proliferation and inflammation^2^. Unfortunately, while DES reduced ISR incidence, it introduced complications such as delayed endothelial healing and increased thrombosis due to the antiproliferative effects of these drugs on endothelial cells^3^.

In response to these limitations, research has focused on developing biodegradable metallic implants^4,5^. These devices offer temporary mechanical support and gradually resorb after fulfilling their purpose, thereby reducing long-term complications linked to permanent metallic backbones. Additionally, they can be combined with local drug-delivery strategies if necessary, addressing both early and late issues seen in current DES technologies. Iron and its alloys, particularly FeMn-based systems, have emerged as promising candidates for stent applications due to their favorable mechanical properties, biocompatibility, and controlled degradation rates^6,7^. Fe–30Mn–1C and a modification with sulfur, fabricated via casting and additive manufacturing, respectively, have shown considerable promise^7–9^. This alloy demonstrates enhanced corrosion rates and superior mechanical strength compared to pure iron, making it highly suitable for stent applications. However, innovative drug delivery systems are necessary to fully address the challenges associated with stent technology.

In addition to iron-based systems, magnesium and zinc alloys have garnered interest as candidates for biodegradable stents due to their favorable corrosion behavior and biocompatibility. While magnesium alloys are biocompatible, they often degrade too quickly to maintain mechanical integrity throughout the healing process^10^. On the other hand, zinc-based alloys offer intermediate degradation rates but may lack sufficient mechanical strength^11^. Although our electrochemical coating strategy was optimized for FeMnC, it could be adapted for other biodegradable metals, including Zn-based systems, as long as appropriate passivation strategies are utilized.

Conducting polymers (CP), particularly polypyrrole (PPy), have gained attention as potential stent coatings with drug-eluting properties. Recent studies have shown that PPy, electrodeposited via chronoamperometry, can modulate the degradation rate of iron and serve as an effective matrix for drug incorporation, including agents like salicylate or dexamethasone^12–14^. The electrodeposition technique facilitates uniform coating on the metallic surface and allows for simultaneous drug embedding, offering advantages over traditional dip-coating methods, which can restrict initial drug release^14^. However, the electrodeposition of PPy coating on active biodegradable metals, the simultaneous incorporation of drug molecules, and ensuring drug-safe and targeted delivery remain challenging.

Nanodiamonds (NDs) have recently attracted attention in medical applications due to their unique properties, including small size, surface functionalization potential, and biocompatibility^15^. Nanodiamonds have a large surface area that can be functionalized with various chemical groups (e.g., carboxyl, hydroxyl, or amine groups) or biomolecules (e.g., antibodies, peptides, or drugs)^16^. This enhances their versatility for targeted delivery, imaging, or therapeutic applications. They have shown promise as drug carriers, minimizing side effects on surrounding tissues and demonstrating efficacy in cancer therapy, antibiotic delivery, and gene therapy^17^. Recent studies have highlighted the potential of nanodiamond-functionalized coatings for electrochemical sensors and biosensors^18^.

While NDs are widely recognized for their biocompatibility and low toxicity, concerns about their release into the bloodstream must be considered. Instead of using NDs as free nanoparticles, electrochemical immobilization into the PPy structure is expected to reduce the risk of detachment or systemic release significantly. Moreover, previous studies have shown that surface-functionalized NDs exhibit a limited inflammatory response and favorable interactions with endothelial and immune cells, supporting their safe use in vascular applications^19^. Functionalization strategies for nanodiamonds continue to evolve, with recent work demonstrating superhydrophobic silane-modified NDs for applications ranging from protective coatings to biomedical devices^20^.

Some studies in the literature on combining CP with nanodiamonds were found. Still, the experiments were primarily focused on pristine NDs, unnecessarily incorporated into CP structure, and noble metals, as in the work of A. I. Gopalan et al.^21^, before polyaniline electrodeposition, the layer of NDs was drop-cast onto a gold electrode. PPy was successfully combined with NDs by chronopotentiometry in the presence of oxalic acid onto stainless steel under ultrasound irradiation^22^.

This study aims to test the hypothesis that (1) one-step electrodeposition of a PPy coating on the surface of a cast biodegradable Fe-30Mn-1C (FeMnC) alloy is feasible, and (2) nanodiamonds can be successfully incorporated into the polymer matrix during this process without altering the alloy’s corrosion properties.

## Results and discussion

In the first step of the study, the nanodiamonds were functionalized with carboxylic (-COOH) and hydroxylic (-OH) groups to enhance their hydrophilicity, thereby improving colloidal stability in the aqueous environment, which is necessary for effective electrodeposition. Moreover, the additional ND groups provide versatile attachment points for other biomolecules (e.g., possible drugs, proteins, peptides), enabling targeted drug delivery or other therapeutic applications.

**Fig. 1:**
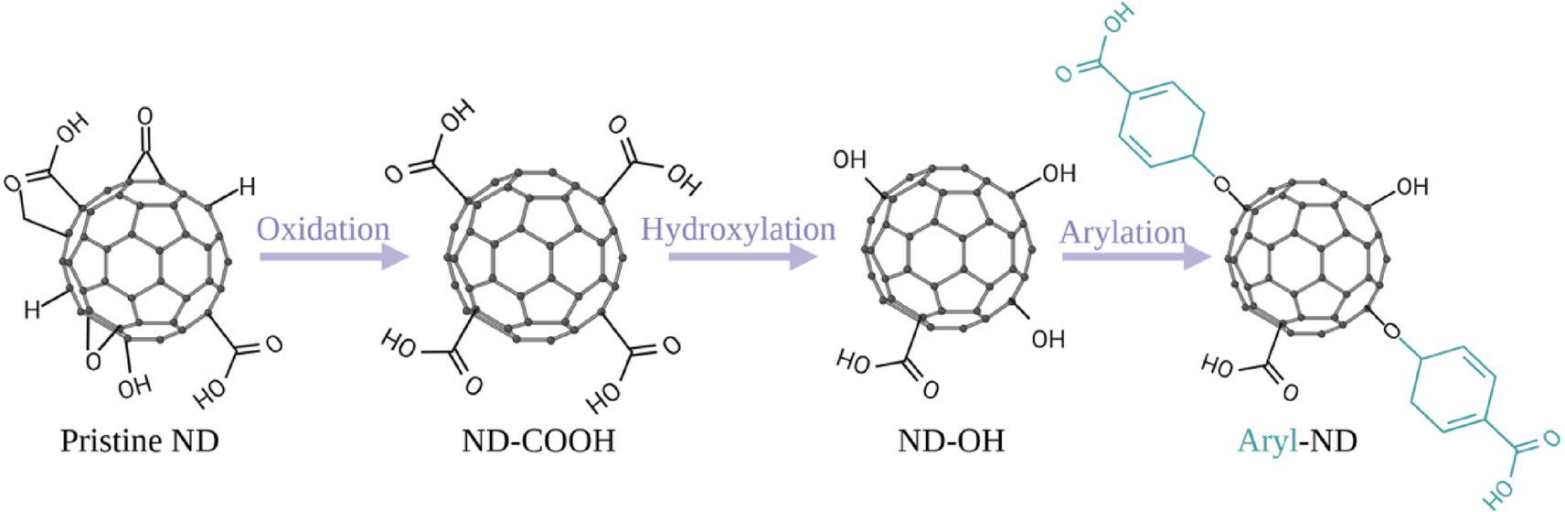
Schematic representation of functionalization steps of ND (created with BioRender).

Pristine NDs were modified in the initial state by high-temperature oxidation and hydroxylated by the borane reduction method. High-temperature oxidation of nanodiamonds introduces oxygen-containing functional groups, primarily carboxyl (COOH) groups, along with aldehydes, ketones, ethers, and alcohols (Fig. 1). Hydroxylation using borane reduces carbonyl groups, such as ketones (C=O), to hydroxyl groups, facilitating subsequent chemical modifications like amidation, esterification, acylation, or silanization. These functionalization steps significantly enhance the versatility of nanodiamonds for various biomedical and chemical applications^23^. Finally, aromatic groups were attached to the surface by arylation. Each fabrication step was described in detail in the SI (Figs. S1–S3).

**Fig. 2:**
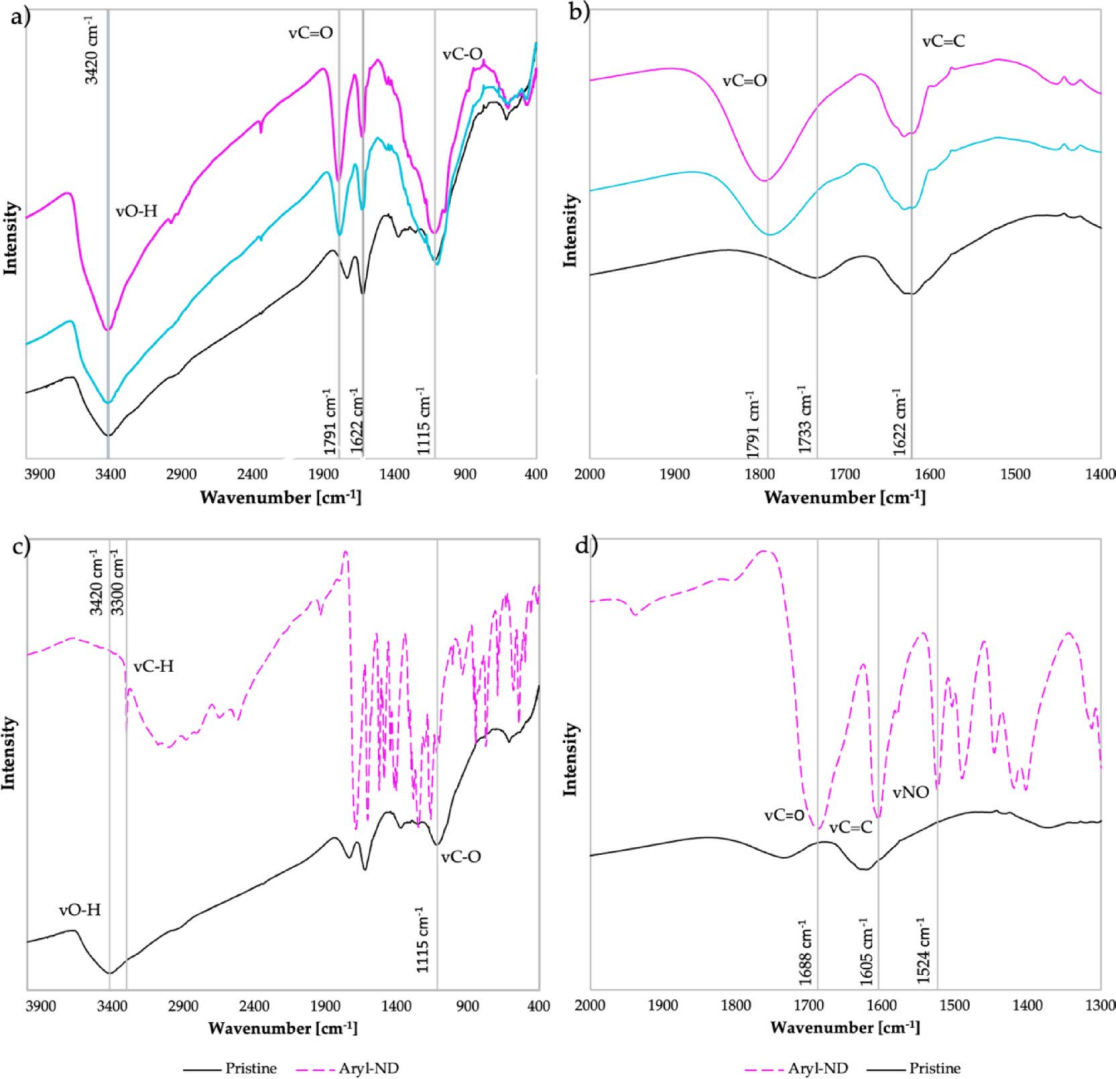
(**a, b**) FTIR spectra of pristine (black), oxidized (pink), and hydroxylated (blue) NDs with different magnifications, (**c, d**) FTIR spectra of pristine (black) and arylated ND (purple) with varying ranges of wave numbers.

The FTIR spectra of NDs were analyzed after each functionalization step (Fig. 2).

The spectra show that oxidized and hydroxylated NDs display characteristic O–H, C=C, and C–O stretching vibrations at 3420 cm⁻^1^, 1622 cm⁻^1^, and 1115 cm⁻^1^, respectively. A pronounced carbonyl (C=O) peak at 1791 cm⁻^1^ is observed, suggesting the presence of aldehydes, ketones, or carboxyl groups. The intensity of this peak increases after oxidation and hydroxylation, indicating successful surface modification. Arylated NDs (Aryl-ND) show a complete disappearance of the O–H and an apparent reduction in C–O peaks, indicating that aryl groups successfully replaced surface hydroxyl groups during the functionalization process^24^. This substitution is further supported by C–H stretching at 3300 cm⁻^1^ and aromatic C=C stretching at 1605 cm⁻^1^.

Additionally, peaks at 1688 cm⁻^1^, 1524 cm⁻^1^, and 1400 cm⁻^1^ correspond to aromatic acid, nitro (–NO_2_), and C–OH groups, respectively, which are characteristic of the arylation process. The band at 1524 cm⁻^1^ is attributed to the asymmetric stretching of –NO_2_ groups formed during diazonium-based functionalization, as reported in previous studies on nitrobenzene-diazonium-modified nanodiamonds^25^.

A detailed summary of the FTIR peak assignments for all analyzed ND samples is provided in Table S1 in the SI, including characteristic bands attributed to aryl functionalities.

**Fig. 3:**
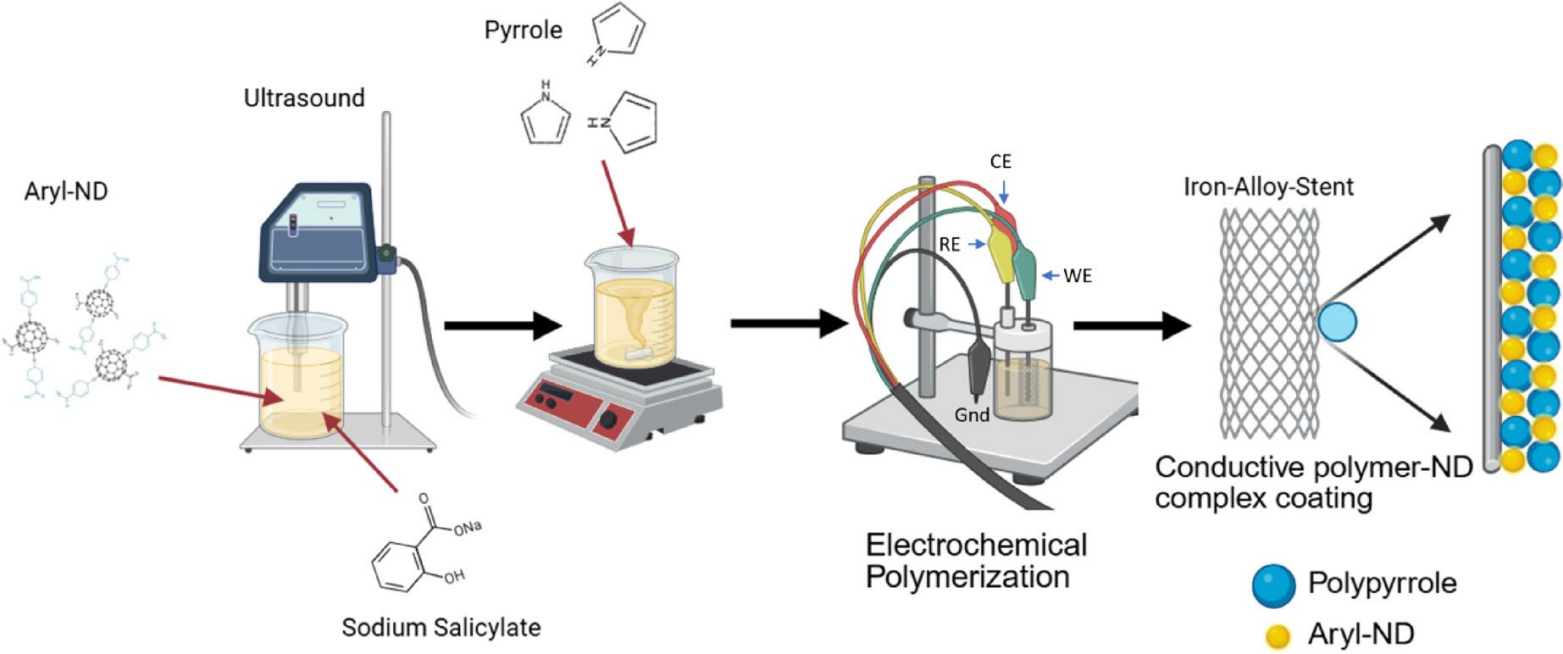
Schematic representation of one-step fabrication of PPy coating doped with Aryl on FeMnC alloy (Gnd—ground, WE—working electrode, CE—counter electrode, RE reference electrode).

These results demonstrate the successful incorporation of functional groups onto the ND surface. Changes in vibrational frequencies indicate that each step modifies the surface groups, supporting the hypothesis that these methods effectively alter ND surfaces for enhanced functionality. The resulting functionalized NDs were characterized with a significantly reduced mean hydrodynamic diameter (HD) of 135 nm and zeta potential (EZ) of − 40 mV, compared to unmodified ND (HD 170 nm, E_z_ +15 mV) (Fig. S4), allowing the formation of stable NDs-water based colloid with a negative charge required for effective electrodeposition. Figure 3 presents a schematic representation of the one-step fabrication of a PPy coating with incorporated Aryl-ND (PPy-ND) on the surface of a biodegradable FeMnC alloy.

**Fig. 4:**
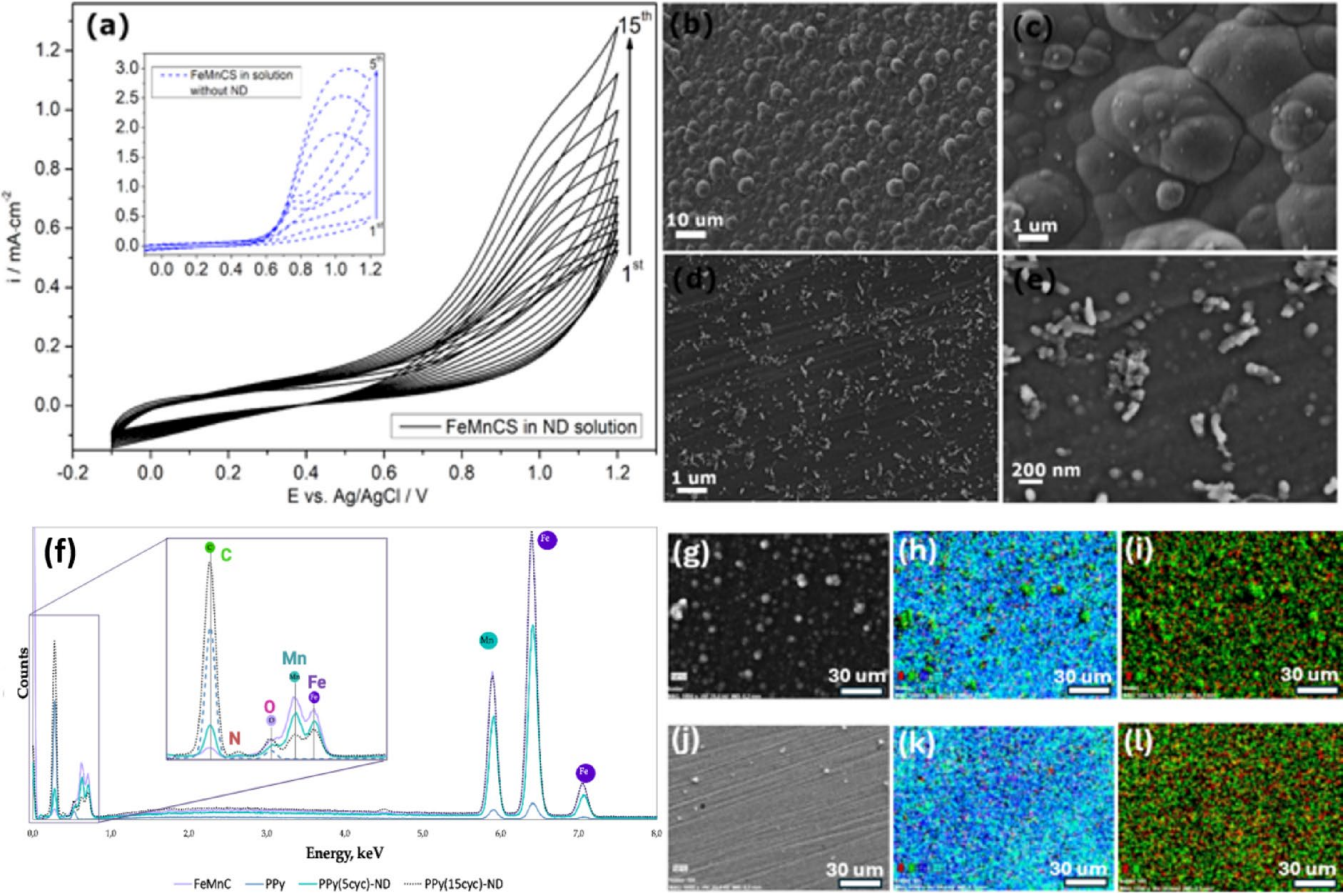
(**a**) Cyclic voltammograms recorded during electrodeposition in an aqueous solution of pyrrole, sodium salicylate, and NDs (inset: without NDs), SEM images of PPy on FeMnC synthesized without NDs (**b, c**) and in the presence of NDs (**d, e**); EDX spectra of FeMnC substrate, FeMnC coated with PPy and PPy with ND after 5 and 15 cycles (**f**)**,** and corresponding EDX mappings of PPy (no NDs) (**g**–**i**), and PPy-ND (**j**–**l**) (Fe-blue, Mn-turquoise, N-red, C-green).

Pyrrole monomer and passivating dopant sodium salicylate were added to the as-prepared colloid of NDs (0.5 mg/ml), further serving as a deposition solution. The concentration of each solution element and deposition parameters were chosen based on the optimization procedure (described in SI). Finally, PPy was deposited on the surface of cast FeMnC by cyclic voltammetry (CV) technique within the potential range of  − 0.1–1.2 V vs. Ag/AgCl with different numbers of cycles. For comparison, PPy was also deposited on FeMnC under the same conditions but without NDs (PPy). Figure 4a presents the voltammograms recorded during the deposition process, highlighting the distinct influence of nanodiamonds on the system. The recorded currents were significantly lower in the presence of NDs, suggesting a less efficient deposition process. This behavior can be attributed to the slower incorporation of both NDs and salicylate into the coating. In particular, the significantly larger size and lower diffusivity of surface-functionalized nanodiamond particles compared to small-molecule dopants (e.g., salicylate or dexamethasone) result in limited mobility and hindered transport toward the growing polymer layer. As negatively charged dopant counter-anions, NDs participate in the charge compensation process during pyrrole oxidation; however, their steric hindrance and reduced diffusion rates slow the polymerization kinetics and reduce current densities. Similar kinetic limitations were previously reported for other bulky or macromolecular dopants incorporated into PPy or polyaniline matrices^26,27^, including salicylate and dexamethasone^14^.

The high zeta potential (approximately − 40 mV) measured for the functionalized NDs supports the formation of a stable colloidal suspension with minimal aggregation, enabling uniform ND distribution in the deposition solution during electropolymerization. Stirring was deliberately avoided, as it is known to destabilize the current response and polymer growth under potentiostatic conditions. The high colloidal stability of the arylated ND dispersion ensures their consistent presence during electropolymerization, which, according to the charge-compensation mechanism of PPy growth, promotes uniform incorporation of NDs into the polymer matrix. A similar incorporation process was previously observed for dexamethasone (DEX), a negatively charged small molecule, during one-step PPy electrodeposition on pure iron surfaces, confirming the effectiveness of this electrostatic-driven approach^14^.

The successful deposition of coatings on the FeMnC alloy was confirmed by SEM (Fig. 4b–e), EDX spectra (Fig. 4f), and EDX elemental mapping (Fig. 4 g–l). A characteristic cauliflower-like morphology of the PPy coating was observed when pyrrole was electrodeposited in the presence of salicylate alone (Fig. 4b, c). The structure became less pronounced for PPy synthesized in the presence of both NDs and salicylate, with additional elongated PPy “sticks” evident (Fig. 4d, e). Such an untypically rough structure was also observed in the chronoamperometric deposition of PPy-oxalic acid-NDs on gold^22^. The incorporation of arylated nanodiamonds into the electropolymerization system significantly influences the kinetics of PPy film growth. Because they are larger and less mobile than small-molecule dopants, NDs serve as slowly migrating counter-ions, which can locally modify the rate of monomer oxidation and polymer chain propagation during deposition. These kinetic effects are known to impact the nucleation density and growth direction of PPy, potentially encouraging the development of more anisotropic or elongated surface structures.

Notably, electrosynthesis of the coating was not feasible in the presence of NDs alone. The active FeMnC alloy requires the presence of a specific salt in the deposition solution to facilitate CP deposition by preventing metal dissolution through surface pre-passivation. In this context, recent studies identified the salicylate-passivating layer during the electrodeposition of PPy on pure iron surfaces^28^. The EDX spectral analysis (Fig. 4f) revealed distinct elemental compositions across the samples. For the bare substrate, prominent peaks of iron (Fe) and manganese (Mn) were observed, while signals from carbon (C) and nitrogen (N) were negligible due to the absence of a coating. Although the FeMnC alloy contains carbon as an alloying element, the C signal was minimal in the EDX spectrum because it is distributed throughout the bulk and there are no surface-exposed carbon-containing layers. Furthermore, the lower sensitivity of EDX to light elements, like carbon, could explain the weak signal observed on the bare substrate. In contrast, the Fe and Mn peaks were significantly diminished when PPy was synthesized using salicylate as the sole dopant over 5 CV cycles, along with a marked increase in the relative intensities of C. Although the nitrogen signal in the spectra is relatively weak due to nitrogen’s low atomic number and the thinness of the deposited films, its presence is further verified by EDX elemental mapping, which displays a uniform nitrogen distribution across the coated substrates. This mapping provides a more dependable confirmation of nitrogen incorporation than isolated spectral peaks alone.

Incorporating nanodiamonds into the PPy coating altered the deposition process, requiring additional time and resulting in higher residual Fe and Mn content after 5 CV cycles. Although the carbon and nitrogen peaks became more pronounced, their intensities were lower than those of pure PPy. Increasing the number of CV cycles resulted in the formation of a thicker PPy coating embedded with nanodiamonds, as evidenced by the stronger C and N signals in the spectrum. The identification of these nanostructures as PPy is supported by EDX mapping and spectra (Fig. 4f–l), which confirm the presence of carbon and nitrogen signals in the regions corresponding to surface features. In contrast, the signals from the underlying FeMnC substrate (Fe, Mn) are diminished. Increasing the number of CV cycles further boosts the C and N signal intensity, consistent with the growth of the polymer layer thickness. EDX mapping (Fig. 4g–l) further confirmed the uniform distribution of the coatings over the substrate in each of the studied cases.

The presence of the PPy coating with incorporated NDs was further confirmed by ATR-FTIR analysis (Fig. S5, Table S2). The spectrum displays characteristic bands of polypyrrole: a broad band near 3300 cm⁻^1^ indicating =C–H stretching vibrations, a peak around 1450 cm⁻^1^ due to C=C stretching in the conjugated backbone, and a band at about 1100 cm⁻^1^ assigned to C–N stretching^29^. Additional bands near 1700 cm⁻^1^ may indicate N–C=O vibrations, while the low-frequency region around 750 cm⁻^1^ corresponds to N–H bending. These features confirm the formation of the PPy layer^12^. Furthermore, signals between 1200–1300 cm⁻^1^, absent in the spectrum of PPy without NDs, support the incorporation of aryl-functionalized nanodiamonds, likely from vibrational modes of the aryl groups^30^. These signals are absent in the spectrum of PPy without NDs, indicating that no other chemical additives were added during synthesis, which supports the assignment of these signals to aryl-specific vibrational modes present on the ND surface.

Although direct visualization of nanodiamonds within the PPy matrix is not possible at SEM imaging scales (Fig. 4d–e), their presence is supported by complementary characterization techniques. EDX elemental mapping confirmed a uniform distribution of carbon and nitrogen across the coating area, while FTIR spectra showed characteristic bands of aryl-functionalized NDs. Along with the previously discussed charge-compensation mechanism and high colloidal stability of the dispersion, these results strongly support the effective and uniform incorporation of NDs into the PPy matrix during electrodeposition.

The composition of the deposition solution, containing passivating sodium salicylate and negatively charged arylated nanodiamonds, facilitated the electropolymerization of PPy on the surface of the active FeMnC alloy. During pyrrole oxidation, the positively charged PPy⁺ chains were electrostatically compensated by the arylated NDs, which acted as counteranions, leading to their incorporation into the growing polymer network. This follows the established charge-compensation mechanism of PPy formation, previously described in detail for salicylate and DEX-based systems on pure iron surfaces^13,14^. In our case, electrodeposition was performed under standard (non-ultrasound-assisted) conditions, yielding uniform PPy coatings as observed by SEM. Although Ashassi-Sorkhabi et al.^22^, used ultrasound-assisted deposition, the key variable in their study was the presence of nanodiamonds, which caused the formation of Cu₂O surface crystals with different sizes and orientations, highlighting the ability of NDs to influence nucleation and film morphology. While direct visualization of NDs within the PPy matrix is not feasible at this scale, the combination of high colloidal stability (ζ ≈ − 40 mV), EDX mapping showing a homogeneous elemental distribution, and FTIR signals indicating the presence of aryl groups provides indirect but converging evidence for their effective incorporation.

**Fig. 5:**
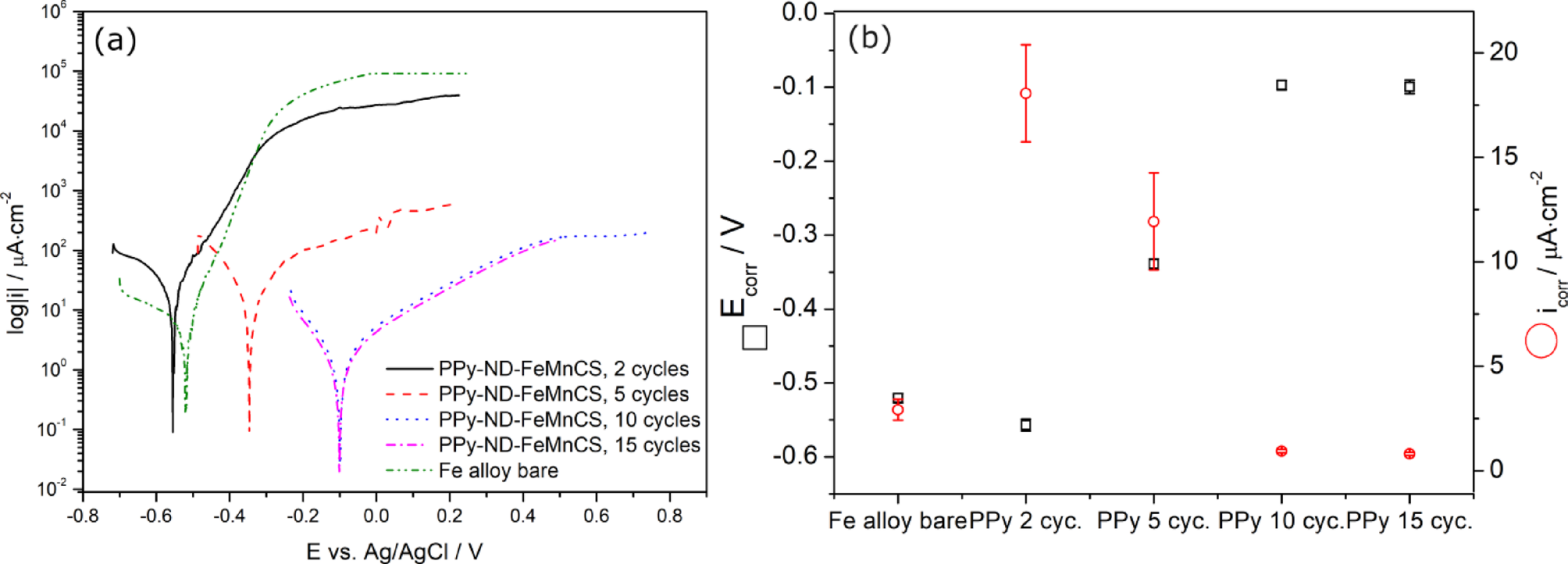
(**a**) Tafel plots, and (**b**) evolution of E_corr_ and i_corr_ of the samples with indicated relative error bars recorded in SBF at 37 °C. Detailed information regarding statistics on corrosion tests is provided in SI, and the results are presented in Fig. S6.

Given the biodegradable application, the materials’ preliminary corrosion properties were evaluated in a simulated body fluid (SBF) solution at body temperature. Fig. 5a, b illustrate the Tafel plots representing the corrosion performance of PPy-ND coatings synthesized with different cycle numbers, along with the corresponding evaluation of corrosion potential (E_corr_) and corrosion current density (i__corr_), respectively.

Adjusting the number of CV cycles, and consequently the thickness of the polymer coating, significantly influenced the corrosion performance of the material. By varying the cycle numbers during the deposition process, it was possible to tailor the metal’s corrosion potential (E_corr_) and current density (i_corr_). Notably, this approach enables the synthesis of PPy coatings incorporating NDs with corrosion rates that can be adjusted to match, exceed, or remain lower than the metallic substrate. This capability offers a promising strategy for tailoring the degradation behavior of metals, addressing a critical and unresolved challenge in the application of degradable stents.

Additionally, a 24-h immersion test in SBF at 37 °C showed that the PPy–ND coating kept its shape after exposure (Fig. S7, Supporting Information), indicating initial stability under physiological conditions. The coating stayed intact, with no visible peeling or surface damage, although some cracking was seen, likely due to drying-induced shrinkage. While this short-term qualitative test cannot predict long-term degradation patterns, previous studies on similar PPy-based systems suggest that the degradation process may involve a redox-coupled reaction between the conducting polymer and the underlying active metallic substrate^12^. In this process, the electrolyte seeps into the porous PPy structure, reaching the Fe-based surface and initiating metal oxidation, which then drives the electrochemical reduction of the PPy coating, resulting in dopant release and coating degradation. The rate and uniformity of degradation can be adjusted by changing the electropolymerization parameters, dopant chemistry, and coating structure. Although this mechanism was not directly tested in this study, it is likely relevant to the PPy–ND coatings here due to their similar structural and electrochemical features. Longer-term degradation studies under dynamic physiological conditions (e.g., 1–3 month immersion in a simulated venous flow model) are planned as future work and will be reported later.

This study demonstrates the successful electrodeposition of the conducting polymer PPy on the surface of an active FeMnC alloy for potential temporary implant applications. It also establishes an effective chemical functionalization procedure for pristine NDs, yielding a highly stable water-based suspension compatible with electrochemical deposition. The combination of passivating salicylate and pyrrole monomer enabled the formation of PPy coatings with embedded NDs on the FeMnC surface in a single-step process. While drug incorporation and release were not addressed in this work, it provides a material and methodological foundation for future drug delivery applications, where NDs can act as customizable carriers. Our previous studies have confirmed the feasibility of incorporating drugs like salicylate or dexamethasone into PPy coatings (without NDs), and further research will build upon the current system to integrate therapeutic agents onto the ND surface. Future studies will also investigate the long-term stability and the impact of PPy-ND coatings on FeMnC degradation kinetics.

## Methods

All experimental procedures were described in detail in the Supporting Information.

## Supplementary Information

Below is the link to the electronic supplementary material.

Supplementary Material 1

## Data Availability

The datasets generated and/or analyzed during the current study are available upon reasonable request from the corresponding authors. Please contact Karolina Cysewska (karolina.cysewska@pg.edu.pl) or Dr. Natalia Beshchasna (natalia.beshchasna@ikts.fraunhofer.de).
