# Supplementary material for: Electro-assisted integration of nanodiamonds into conducting polypyrrole for functional coatings
Source: Sci Rep. 2025 Dec 13;16:1808. doi: 10.1038/s41598-025-31402-6 (PMC12804897; doi:10.1038/s41598-025-31402-6)
Supplement: Supplementary file 1 — Supplementary Material 1 [file 41598_2025_31402_MOESM1_ESM.docx]

**SUPPORTING INFORMATION**

**Electro-Assisted Integration of Nanodiamonds into Conducting Polypyrrole for Functional Coatings**

Karolina Cysewska^1,^ *, Anita Stoppel^2^, Muhammad Saqib^2^, Birgit Paul^3^, Julia Kristin Hufenbach^3^, Joerg Opitz^2^ and Natalia Beshchasna^2,^ **

**1. Materials and methods**

**a)** Fabrication of FeMnC

Pure elemental constitutes were melted in an Al_2_O_3_ crucible under argon atmosphere in an induction furnace (Balzer) and subsequently cast into a copper mould to receive the Fe69Mn30C1 (in wt%; FeMnC) ingot. Cast FeMnC samples were polished with SiC abrasive papers up to 2000 grid, rinsed with ethanol, and dried in the air.

**b) Preparation of functionalized NDs**

- Oxidation of Nanodiamonds (ND-COOH)

The high-temperature air oxidation method introduced a homogeneous layer of carboxylic (COOH) groups on the nanodiamond surface. For this process, approximately 800 mg of untreated nanodiamonds (positively charged raw DND Powder, 1.7 % ash, SKU: NDStandard50g, Average size: 170 nm, Shape: spherical clusters/agglomerates) (NDs) (Adamas Nanotechnologies) were evenly distributed in ceramic dishes (diameter: ~6 cm) to avoid the formation of thick powder layers and to ensure uniform exposure to air oxidation (Figure S1a). Multiple dishes were used to maximize surface exposure. The oxidation was performed in an HTK 16/17 furnace at 415 °C for 5 hours with a 20 K/min heating rate in ambient air. While this oxidation process effectively introduced carboxyl groups, some tertiary alcohol functional groups remained inaccessible. The oxidized nanodiamonds underwent a subsequent reduction process to achieve a homogeneous surface functionalization.

- Hydroxylation of Nanodiamonds (ND-OH)

The hydroxylation of nanodiamonds was carried out using a borane reduction protocol^1^. This method effectively converts carbonyl groups to hydroxyl functionalities. In a typical procedure, 2 g of oxidized nanodiamonds (ND-COOH) were suspended in 120 ml of dry tetrahydrofuran (THF) (99.85 %, Life Technologies GmbH, Darmstadt, Germany) in a round-bottom flask. Subsequently, 5 ml of 1.0 M borane-tetrahydrofuran (BH₃-THF) (Sigma-Aldrich Chemie GmbH, Taufkirchen, Germany) solution was added dropwise with continuous stirring. To maintain the system at a stable temperature and prevent boiling, the flask was equipped with a glass condenser and placed over an oil bath at 64 °C under a nitrogen atmosphere. The reaction was allowed to reflux for 24 hours.

After cooling to room temperature, the reaction mixture was hydrolyzed by adding 2 M HCl (Sigma-Aldrich Chemie GmbH, Taufkirchen, Germany) dropwise until hydrogen evolution ceased, and no air bubbles were visible. The resulting solid product was isolated via centrifugation at 15,000 rpm and washed four times with ultrapure water (ddH₂O) and three times with acetone. Each washing step included 10 minutes of ultrasonication, followed by 30 minutes of centrifugation and replacement of the supernatant with fresh solvent. Before further use, the hydroxylated nanodiamonds (ND-OH) were dried overnight in a vacuum desiccator.

| 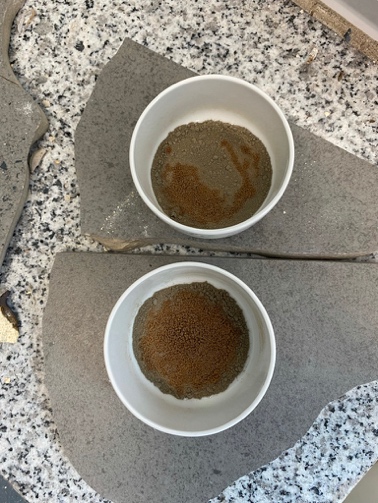  a) | 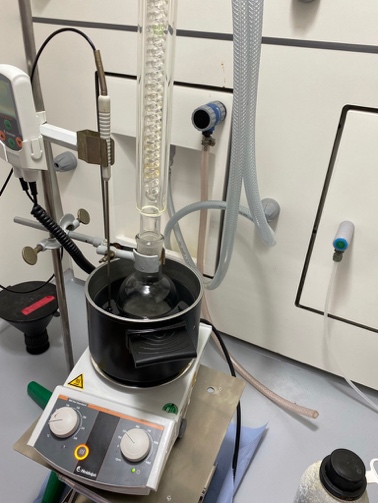  b) | 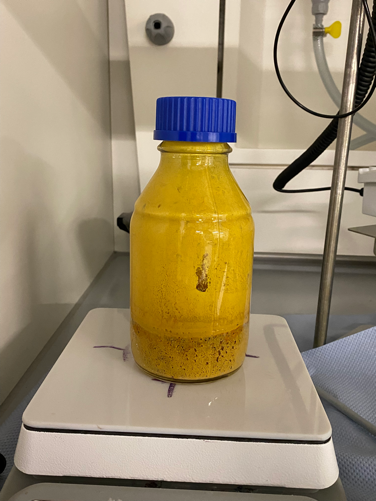  c) |
| --- | --- | --- |

Figure S1. Experimental Setup for a) oxidation, b) hydroxylation, and c) arylation.

- Arylation of Nanodiamonds (Aryl-ND)

Arylation of nanodiamonds was performed via the diazonium salt reaction, where aromatic groups were covalently bound to the ND surface. For this process, 10 g of aminobenzoic acid (>99%, Sigma-Aldrich Chemie GmbH, Taufkirchen, Germany) and 5 mL of amyl nitrite (96 %, Sigma-Aldrich Chemie GmbH, Taufkirchen, Germany) were added to 10 mL of an aqueous suspension containing 1.5 g of nanodiamonds. Amyl nitrite facilitated the in-situ generation of diazonium salts^2^, which were subsequently conjugated to the NDs via click chemistry^3^. The reaction mixture was stirred at 80 °C on a hot plate for 15 hours (Figure S1c).

- Washing of functionalized Nanodiamonds (NDs)

After the reaction was completed, the suspension was cooled to room temperature, and a thorough washing procedure was implemented. The protocol involved up to five wash cycles with ultrapure water and 15 cycles with acetone to achieve a transparent supernatant, as shown in Figure S2. The washing process ensured the removal of excess aminobenzoic acid and other byproducts. Initially, the supernatant appeared dark red to brownish and gradually became yellow with repeated washing, achieving transparency.

The washed nanodiamonds were dried overnight in a vacuum desiccator. Once dried, the arylated nanodiamond powder was ground using a marble pestle to break up agglomerates, ensuring a fine and homogenous powder for subsequent experiments.


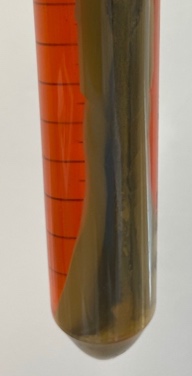

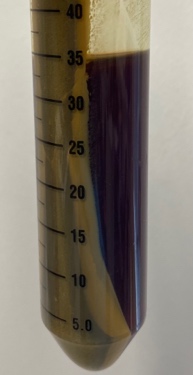

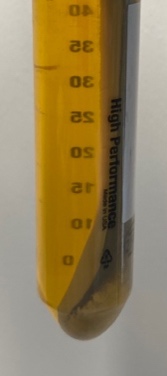

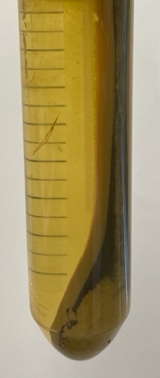

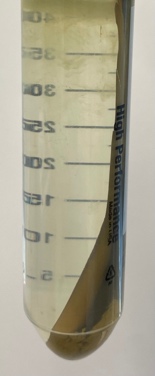

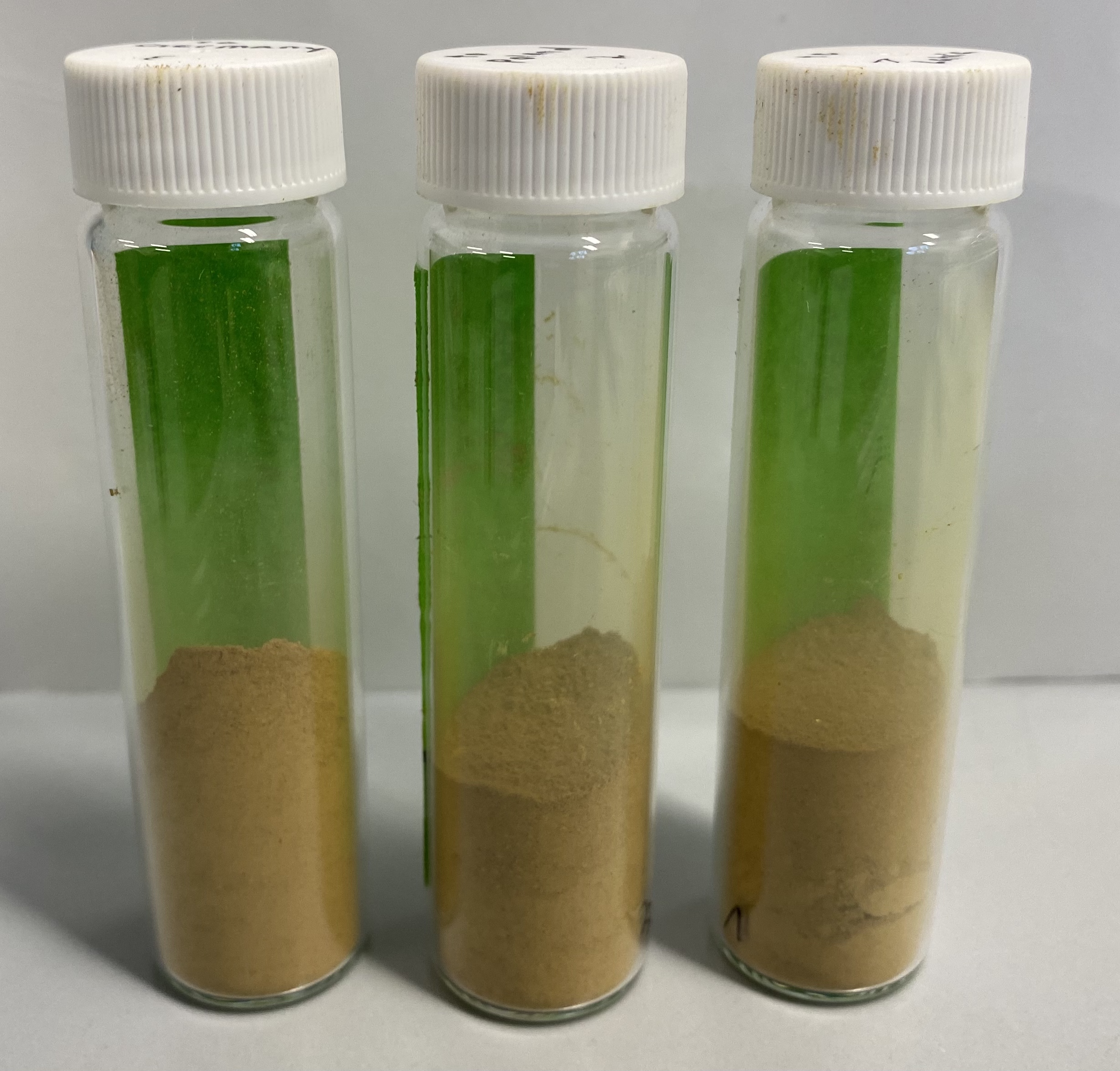


**Figure S2.** Arylated NDs and supernatant after centrifugation from left to right: after one, two, five, ten, and twenty cycles of washing; last: dried arylated ND powder.

In the next step, 50 mg of arylated NDs were dispersed in 50 ml of ultrapure water. The solution was sonicated with a Sonopuls HD 2200 ultrasonic homogenizer (Bandelin Electronic GmbH and Co.) for 30 min at 20 kHz and 70 % of the device power in a pulsed cycle with 0.7 s active and 0.3 s passive pauses to deagglomerate the NDs powder. The structure of Aryl-ND after each washing step was monitored by Fourier Transform Infrared Spectroscopy (FTIR; FTIR-6100PerkinElmer, Rodgau, Germany) (Figure S3).


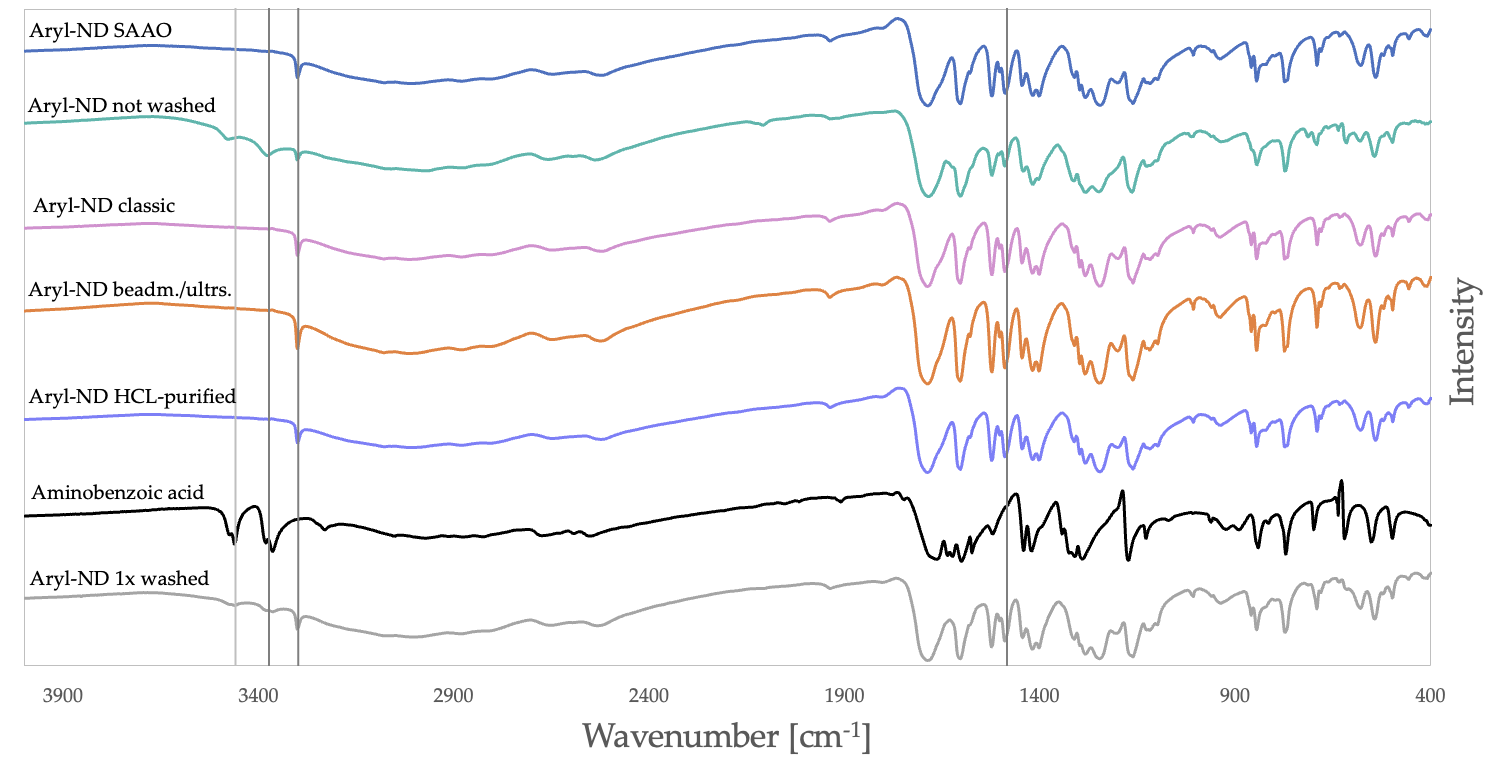


**Figure S3.** FTIR spectra of Aryl-ND in different washing stages with different deagglomeration treatments before Arylation in the 400-4000 cm^-1^ range compared to pure aminobenzoic acid.

**Table S1.** Characteristic bonds of ND samples corresponding to their wavenumbers (FTIR analysis).

| Figure | Wavenumber (in cm^-1^) | Functional Group | Possible Interpretations |
| --- | --- | --- | --- |
| 3a | 3420 | O-H stretching (hydrogen-bonded) | Indicates surface hydroxylation, typical of alcohol groups formed during oxidation or hydroxylation. |
| 3a, 3b | 1791 | C=O stretching | Suggests presence of high-frequency carbonyl band; could correspond to acid halides or strained carbonyl species if formed during oxidation/chlorination. |
| 3b | 1733 | - | Likely C=O stretching from ester or carboxylic acid; needs confirmation based on context or additional peaks. |
| 3a,3b | 1622 | C=C stretching | Indicates aromatic or cyclic alkene structures, possibly from graphitic or arylated surfaces. |
| 3a | 1115 | C-O stretching | Suggests ether linkages, possibly from surface-bound alkoxy groups or residual solvents. |
| 3c | 3420 | O-H stretching (hydrogen-bonded) | Confirms hydroxylation, consistent with surface modification or arylation with hydroxylated groups. |
| 3c | 3300 | ≡C–H stretching | Indicates presence of terminal alkynes, possibly introduced via arylation with ethynyl groups. |
| 3d | 1688 | C=O stretching | Suggests conjugated carbonyl groups, often found in aromatic acids or ketones. |
| 3d | 1605 | C=C stretching | Supports presence of aromatic rings, consistent with arylation. |
| 3d | 1524 | N-O stretching | Indicates nitro groups, possibly introduced via arylation with nitrobenzene derivatives. |
| 3c | 1115 | C-O stretching | Reaffirms ether linkages, possibly from arylated or hydroxylated surface groups. |


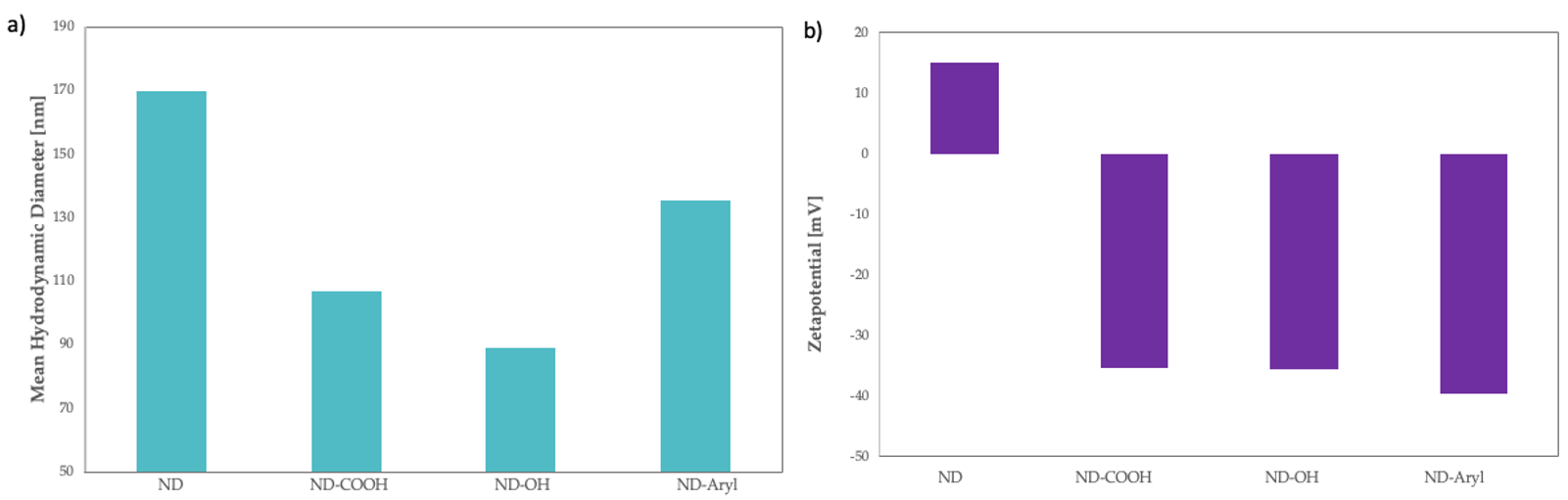


**Figure S4.** a) Mean hydrodynamic diameter and b) Zeta potential of ND in different functionalization stages.

**c) Synthesis of PPy coatings**

Polypyrrole (PPy) coatings were electrosynthesized in an aqueous solution containing 0.1 M sodium salicylate and 0.1 M pyrrole, with or without NDs (0.5 mg ml^-1^) using cyclic voltammetry (CV). The selected synthesis conditions, including compound concentration, electrodeposition technique, potential range, and cycle number, were optimized to achieve well-deposited coatings.

The electrodeposition process was conducted in a three-electrode, water-jacketed cell controlled by a potentiostat Autolab PGSTAT204 (Metrohm, Herisau, Switzerland). The working electrode was bare FeMnC with an exposed surface area of 0.11 cm^2^. An Ag/AgCl electrode in saturated KCl was used as the reference electrode, and a platinum grid (BioLogic) served as the counter electrode. The temperature during the process was maintained at 23 °C +/- 1 °C.


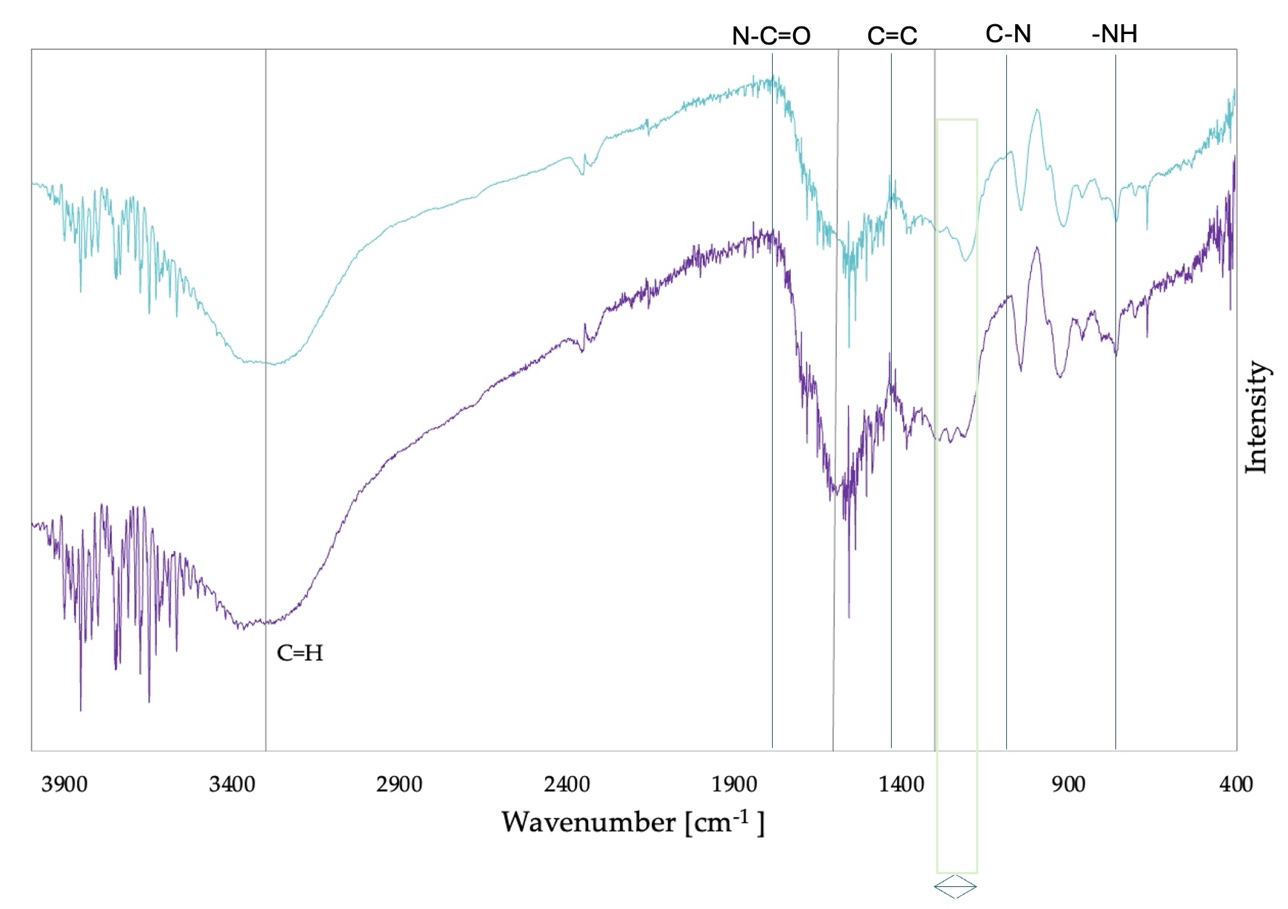


**Figure S5.** ATR-FTIR spectra of PPy (upper) and PPy-ND (lower) coatings.

**Table S2.** Characteristic bonds of PPy, corresponding to their wavenumbers (FTIR analysis).

| Bond | Wavenumber / cm^-1^ | Assignment |
| --- | --- | --- |
| -NH | ~750 | N–H out-of-plane bend |
| C-N | ~1100 | C–N stretching |
| C=C | ~1450 | Ring stretching in PPy |
| N-C=O | ~1700 | Possible carbonyl groups |
| C=H | ~3300 | Aromatic =C–H stretching |

**c) Morphological and structural analysis**

The morphology and structure of the electrodes were characterized using scanning electron microscopy (SEM) with energy-dispersive X-ray (EDX) analysis. SEM imaging was performed with a Zeiss NVision 40 FIB-SEM (Carl Zeiss AG, Oberkochen, Germany) equipped with an XFlash 5010 EDX detector (Bruker AXS, Bruker, Germany). Before analysis, samples were sputter-coated with a thin gold layer to improve conductivity. SEM images were taken at an accelerating voltage of 10 kV and a working distance of 6.5–7 mm. EDX measurements were carried out under the same 10 kV acceleration voltage, with a working distance of 10 mm.

FTIR identified the surface functional groups of NDs measured from 450 cm^-1^ to 4000 cm^-1^ with 4 scan cycles and a resolution of 2 cm^-1^ using a Spectrum Two FT-IR spectrometer in pellets with KBr (1:40 mass ratio of sample to KBr). KBr pellets were prepared by mixing 2 mg of ND and 100 mg of KBr (Sigma-Aldrich Chemie GmbH, Taufkirchen, Germany). The samples were ground with a mortar pestle and placed in a vacuum press. Then, the sample pellet was pressurized manually with a hydraulic press to produce a homogeneous slice of the analyzed sample. Finally, the prepared disc was secured in the device's opening, which had a diameter of 1 cm, and inserted into the spectrometer. Attenuated total reflectance (ATR)-FTIR was used to analyze the chemical composition of the coated samples in as-prepared films-substrate form with a scan rate of 32 and a resolution of 1 cm^-1^ in the wavelength range of 400 to 4000 cm^-1^. All FTIR spectra were baseline-corrected and normalized before analysis, allowing a reliable comparison of absorption bands and relative intensities across all samples.

Nanoparticle tracking analysis (NTA; ZetaView PMX120, Particle Metrix GmbH, Inning am Ammersee, Germany) determined the particle size distribution of NDs. For this purpose, 1 mg of ND was dissolved in ddH_2_O and diluted to the desired concentration. Measurements were recorded at RT and a neutral pH = 7. The laser's wavelength was 488 nm.

**c) Electrochemical measurements**

All electrochemical studies were conducted in a three-electrode, water-jacketed cell controlled by a potentiostat Autolab PGSTAT204 (Metrohm, Herisau, Switzerland). The working electrode was either bare FeMnC or FeMnC coated with PPy or PPy-NDs, with an exposed area of 0.11 cm^2^. The reference electrode was Ag/AgCl in saturated KCl, and the counter electrode was a platinum grid (BioLogic). The measurement temperature was maintained at 37° C +/- 1 °C using a thermostat TC120-ST26 (Grant Instruments, United Kingdom).

Corrosion studies were performed in simulated body fluid (SBF) prepared according to Kokubo and Takadama’s composition (Na^+^ - 142 mmol L^-1^, K^+^ - 5 mmol L^-1^, Mg^2+^ - 1.5 mmol L^-1^, Ca^2+^ - 2.5 mmol L^-1^, Cl^-^ - 148.6 mmol L^-1^, HCO_3_^-^ - 4.2 mmol L^-1^, HPO_4_^2-^ - 1.0 mmol L^-1^, SO_4_^2-^ - 0.5 mmol L^-1^) (*T. Kokubo, H. Takadama, Biomater. 2006, 27, 2907*). Potentiodynamic polarization measurements were performed starting at –0.15 V vs. OCP and scanning up to 1.5 V vs the reference electrode at a rate of 0.5 mV·s^-1^. The OCP of the system was stabilized for a minimum of 15 minutes before each measurement. This protocol follows widely accepted procedures for estimating corrosion parameters such as E_corr_ and i_corr_ using Tafel extrapolation and agrees with international standards (e.g., ASTM G5, ASTM G59) and established literature in the field of electrochemical corrosion testing ^4^.

**Statistics:**

All quantitative measurements were performed with at least three independent replicates (four for corrosion tests). The results are shown in the main manuscript as mean values with error bars indicating standard deviation (SD) or relative error (RE), depending on the figure caption. For corrosion data (E_corr_ and I_corr_), full replicate potentiodynamic polarization curves are provided below to demonstrate reproducibility (Fig. S6). All analysis, including statistical, was performed using OriginPro (OriginLab Corporation), and the results include mean, standard deviation (SD), sum, minimum, median, and maximum values for each dataset. The low relative errors and narrow standard deviations observed confirm the reproducibility of the measurements and the robustness of the dataset used for analysis in the main text.


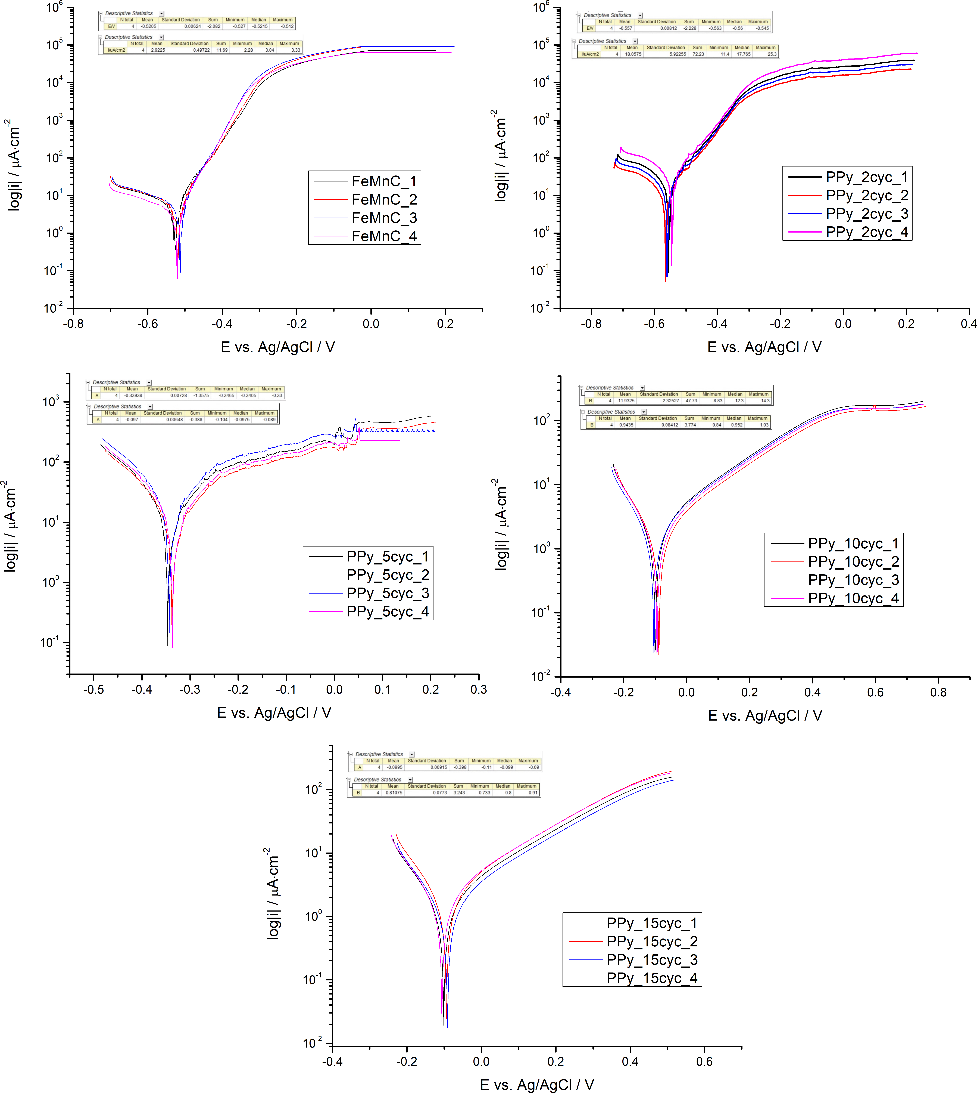


**Fig. S6**. Reproducibility of potentiodynamic polarization measurements in a Tafel representation, for all studied samples in SBF at 37 °C. Each plot shows four independent replicate Tafel curves of potentiodynamic curves recorded under identical conditions (–0.15 V vs. OCP to +1.5 V vs. Ag/AgCl, scan rate 0.5 mV·s⁻¹).

**d) Short-term immersion stability test**

To evaluate the initial stability of the electrodeposited coating under physiological-like conditions, a PPy-ND-coated FeMnC stent was immersed in simulated body fluid (SBF, pH 7.4) at 37 °C for 24 hours. The SBF solution was prepared following the Kokubo protocol and maintained at a constant temperature in a water bath. After incubation, the stent was carefully removed, rinsed with ultrapure water to remove residual salts, and air-dried at room temperature. The morphology of the coating was then assessed using scanning electron microscopy (SEM) (Figure S6). Surface cracks observed after drying were noted and discussed in relation to possible post-immersion shrinkage rather than in-solution degradation.


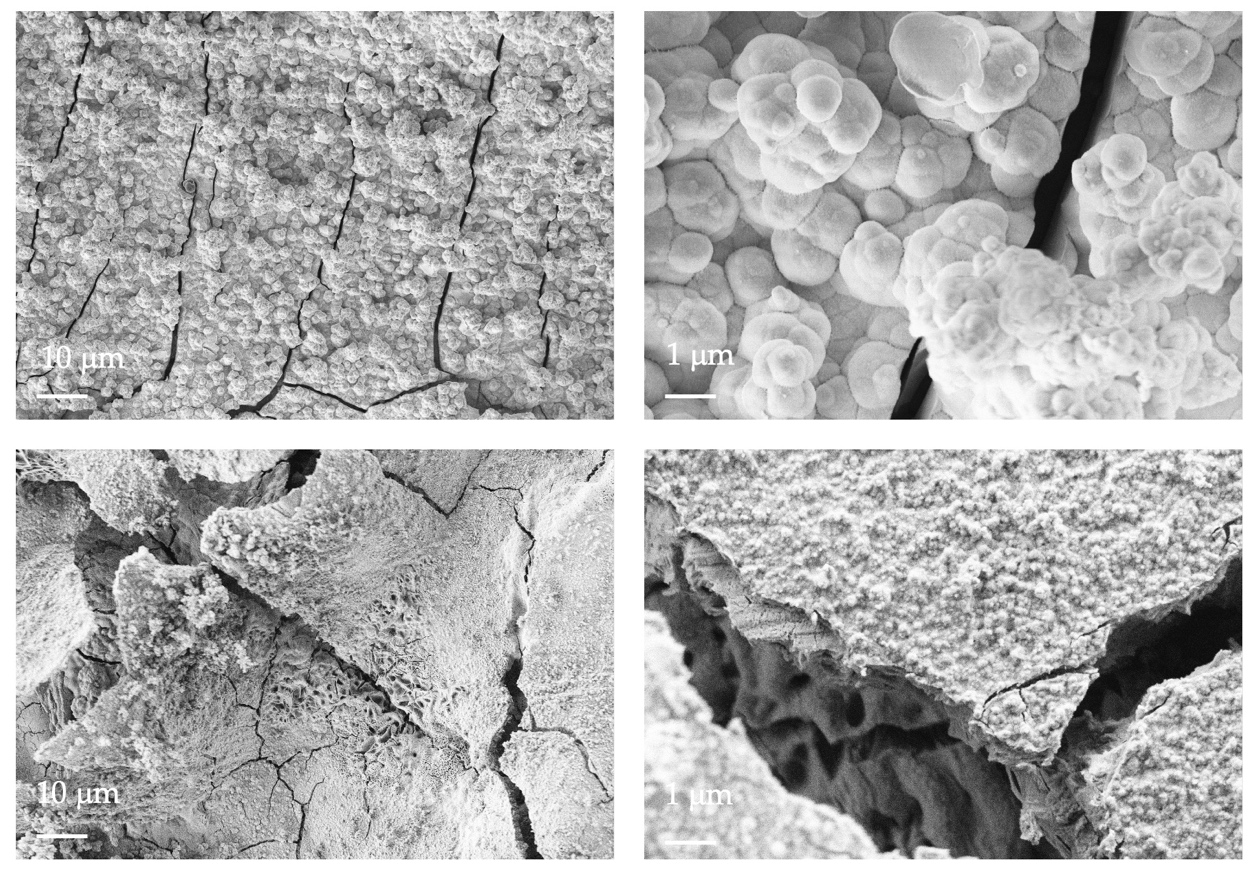


**Figure S7.** SEM images of a FeMnC coated with PPy-NDs after 24 h immersion in simulated body fluid (SBF, 37 °C). 



**References:**

(1) Edgington, R.; Spillane, K. M.; Papageorgiou, G.; Wray, W.; Ishiwata, H.; Labarca, M.; Leal-Ortiz, S.; Reid, G.; Webb, M.; Foord, J.; Melosh, N.; Schaefer, A. T. Functionalisation of Detonation Nanodiamond for Monodispersed, Soluble DNA-Nanodiamond Conjugates Using Mixed Silane Bead-Assisted Sonication Disintegration. *Scientific Reports 2018 8:1* **2018**, *8* (1), 1–11. https://doi.org/10.1038/s41598-017-18601-6.

(2) Krüger, A.; Liang, Y.; Jarre, G.; Stegk, J. Surface Functionalisation of Detonation Diamond Suitable for Biological Applications. *J Mater Chem* **2006**, *16* (24), 2322–2328. https://doi.org/10.1039/B601325B.

(3) Poonthiyil, V.; Lindhorst, T. K.; Golovko, V. B.; Fairbanks, A. J. Recent Applications of Click Chemistry for the Functionalization of Gold Nanoparticles and Their Conversion to Glyco-Gold Nanoparticles. *Beilstein journal of organic chemistry* **2018**, *14*, 11–24. https://doi.org/10.3762/BJOC.14.2.

(4) Brett, C. M. A.; Maria, A. N. A.; Brett, O. *Electrochemistry: Principles, Methods, and Applications*; Oxford Science Publications, 1993.
